# Comparison of the students’ satisfaction about the performance of academic advisors before and after the advisor project in Shahrekord University of Medical Sciences

**Published:** 2014-01

**Authors:** MASOUMEH DELARAM, SARA HOSSEINI

**Affiliations:** 1 Midwifery Departement, Nursing & Midwifery School, Shahrekord University of Medical Sciences, Shahrekord, Iran;; 2 Midwifery Department, Nursing & Midwifery School, Isfahan University of Medical Sciences, Isfahan, Iran

**Keywords:** Advising, Satisfaction, Student, Academic advisors

## Abstract

**Introduction**: Inappropriate advice interferes with the students’ achievement of educational and professional goals and they may fail to use proper resources for their educational needs. The present study was carried out to compare the students’ satisfaction about the performance of academic advisors before and after the advisor project in Shahrekord University of Medical Sciences.

**Methods**: This longitudinal study was carried out using census report on 244 students in different courses at Shahrekord University of Medical Sciences in two stages at the first semester of 2010-2011 and first semester of 2011-2012. To collect the data, we used a self-designed questionnaire including individual and educational information and 10 multiple-choice questions with Likert scale to assess the students’ satisfaction about the advisors’ performance. Data were analyzed in SPSS 14 using paired t-test, qui-square test. P<0.05 was considered significant.

**Results**: Of the ten items of satisfaction, there was only a significant difference in "accessibility to an advisor before and after the advisor project in students of nursing and midwifery school (p=0.010), and the difference was not significant in other items in these students. No significant difference was found in ten items of satisfaction in students at other schools before and after the advisor project (p=0.010).

**Conclusion**: It seems that the implementation of advisor project could not provide a satisfactory position for students. Adequate supervision of university officials on proper implementation of the advisor project, supporting faculty advisors and strengthening their position can help to improve the process.

## Introduction

University students experience more psychological and social stress including separation from the family's previous life, beginning the student life and the need to adapt with the university environment, management of educational, economical and emotional issues due to their sensitivity to the growth and education and the specific conditions at this stage of life, especially at the beginning of entrance to university. With regard to these problems, the necessity of consultation is of great importance. Counseling is comprised of all moral activities in which the counselor tries to help the person to perform activities to solve his/her problems (1). One of the responsibilities of faculty members in addition to teaching and researching is providing the students with counseling. If it is done well, it can help to solve the students’ problems and prevent their academic failure. The responsibility of academic advisors in directing, guiding and supporting students is very effective in achieving the educational goals, especially for those who enter a new environment from far and near villages and towns and are faced with issues such as separation from family, living in a dormitory, and studying in a co-educational environment (2). The students expect that the advisor, as a coordinator, to have sufficient knowledge and information about the curriculum, educational issues, and personal, medical and counseling services (3). Despite the need for counseling, several studies have reported the assessment of student counseling at universities; unfortunately, the students do not consider the teachers as a reliable source of reference to meet their educational needs.  In this regard, a study from Hamedan University of Medical Sciences has reported that the advisors have not been successful in providing advice and guidance to students, especially in medicine and dentistry fields (4). The results of one study in Isfahan University of Medical Sciences have also shown that just one third of the students are satisfied with their supervisor and only thirty-five percents of them are pleased with their supervisors’ sufficient information regarding educational and disciplinary rules (5). Adhami et al. have also reported that the advisors have failed to achieve asatisfactory status among students (6). The findings of a foreign study also reported that the students do not get enough satisfaction from providing advice and guidance to teachers (7). The results of one study suggest that the supervisors´ activities, despite the emphasis on academic system and procedures, have a problem in performance and have not been seriously well received not only by the students, but also by the faculty advisors and it makes supporting and guiding the students difficult (5, 8). With regard to the responsibility of academic advisors in providing advice and counseling to meet the needs of students, the lack of clear information about the status of the services offered at the Shahrekord University of Medical Sciences in Iran and also considering the implication of advisor project at this university, in this study we aimed to compare the level of satisfaction in students from the performance of advisors before and after the implication of advisor project.

## Methods

All the students of medicine, nursing, midwifery, operation room, health, anesthesia, radiology and laboratory sciences were the population of this longitudinal study in four schools of Medicine, Nursing and Midwifery, Health and Paramedical Sciences. This study was conducted in two stages. The first stage of the project was carried out in the first semester of 2010-2011, and before the implementation of advisor project in Shahrekord University of Medical Sciences. The Personal information including age, gender, being native and employment during the education and also their academic information including GPA, level of education, educational field and probation history were assessed by using a self-designed questionnaire. Also the students responded to items such as having or not having an advisor, reason of referral to the advisor, source of educational information, knowing how to ask a counselor for helping , formation or lack of academic records, educational problems due to lack of guidance, and consultation with the advisor about continuing education and his/her response were assessed. Finally, the students' satisfaction about the performance of the advisor was assessed in the form of 10 multiple-choice questions with Likert scale.  It must be mentioned that in the first stage of the project, 300 students including all students studying in the first semester of 2010-2011 were enrolled in the study with census report and completed the questionnaire. After two semesters of their study and performing the advisor project at the university, the research started the second stage of the study that was done in the first semester of 2011-2012, and the same students were tested again with the same questionnaire. At this stage, of the 300 students participating in the first stage, 244 filled out the questionnaire in this stage and the final analysis was performed on the number of 244 subjects. Then, the students΄ satisfaction from the advisors´ performance was compared in two stages. The validity of the questionnaire was determined by the opinion of the faculty and its reliability was confirmed using test-retest (r=0.84). The questions of satisfaction were prepared in Likert scale, and the score for each of the questions about satisfaction could be 1 to 3. Therefore, the maximum score for each factor can be 3, which means high satisfaction of advisor performance and the minimum score can be that means low satisfaction of advisor performance.  Before the distribution of questionnaires among the students participating in the study, the researcher explained the importance of the project, voluntary participation of students, and confidentiality of information and use of them only for research purposes. Because the questions had different options and it was not possible to calculate a total score, the analysis of data was done based on individual questions of satisfaction. For data analysis in SPSS 14 (SPSS Inc, Chicago, IL, USA), descriptive statistics (mean, standard deviation) and analytical statistics (paired t-test, Chi-square test) were used and p<0.05 was considered statistically significant.

## Results

In this study, out of the 300 students enrolled in this study, 244 continued the study by the end of the second stage and completed the questionnaire; therefore, the final analysis was performed on this number. The mean age of the students was 20.25±0.96. About 70% of them were female and 91.4% were single. Grade point average of the students was 15.50±1.30. The highest percentage of students was related to nursing students and the lowest percentage to anesthesiology students. About 90% of them were studying in bachelor degree and 9.4% in MD and DMD programs.  All of the students in this study were studying in day courses and 67.6% of them were native of Chahar-Mahal and Bakhtiari province. In terms of employment during the academic education, 9% of the students were employed and 4.9% of them reported the history of probation in past semesters.  Before the implementation of advisor project 91.4% of the students reported having an advisor and after performing the advisor project 97.2% of them reported having an advisor and the difference was significant (p<0.001). The reason of referral to the adviser was also one of the items which was significantly different before and after performing the project (p<0.001).  So, most of the students stated that the reason of referral to the advisor was for course selection and academic problems.  After performing the advisor project, a significant difference was found in the number of students who called the advisor as a source of their educational information (p<0.001). Before the performing the advisor project, 79.5% and after that 85.2% of the participants knew they can ask an advisor to help them; the difference was significant (p<0.001). Also before the implementation of the project, 60.2% of the students and after that 53.3% of them didn’t know whether the advisor has made the academic records for them or not; the difference was significant (p<0.001). There was not a significant difference in percentage of the students who had difficulty due to inappropriate counseling before and after the implementation of the advisor project [(33.8% vs. 33.2%), p=0.300]. A significant difference was found in the students´ question from the advisor about continuing the education [(27.5% before and 36.9% after the project), p=0.040]. The comparison of mean scores of satisfaction in students of medicine, health, and paramedical school is shown in Tables 1, 2 and 3.  There was no significant difference in any of the ten items of satisfaction before and after the implementation of advisor project. The comparison of mean scores of satisfaction in students of nursing school is shown in Table 4. It was indicated that there was a significant difference only in one of the items of satisfaction called “providing access to an advisor” before and after the implementation of the advisor project (p=0.010).

**Table 1:** Mean scores of satisfaction about the performance of advisor in students of medical school

**Performing the advisor project ** **Students´ satisfaction**	**Before (n=23) Mean±SD**	**After (n=23) Mean±SD**	p
Students’ satisfaction about the program of advisor’ presence at work	0.60±2.60	0.7±2.50	0.290
Devoting sufficient time for counseling	0.70±2.80	0.90±2.50	0.230
Providing access to advisor	1.00±2.70	0.80±2.50	0.320
Student’s benefit from the advice	0.70±2.80	0.80±2.60	0.350
Student’s satisfaction about the impact of counseling	0.70±2.80	0.70±2.80	1
Students’ satisfaction about the advisor for solving the problem	0.90±2.90	0.80±2.90	1
Respectful behavior of advisor with the students	0.80±3.40	0.40±3.80	0.070
The skill of advisor in the students´ problem solving	0.80±3.00	0.80±3.10	0.800
Continuous training of advisor in student's problem solving	2.80±0.80	3.00±0.60	0.090
Friendly relationship between the student and advisor	0.70±3.30	0.60±3.60	0.120

**Table 2:** Mean scores of satisfaction about the performance of advisor in students of Health school

**Performing the advisor project ** **Students´ satisfaction**	**Before (n=22) Mean±SD**	**After (n=22) Mean±SD**	p
Students’ satisfaction with the program of advisor ’ presence at work	0.70±1.80	090±2.00	0.420
Devoting sufficient time for counseling	0.80±1.50	0.60±1.90	0.110
Providing access to advisor	1.00±1.90	1.00±2.30	0.140
Student’s benefit from the advice	1.00±1.00	1.10±2.00	0.210
Student’s Satisfaction about the impact of counseling	1.10±1.20	1.20±1.20	0.290
Students’ satisfaction about the advisor for solving the problem	1.20±1.20	1.20±1.22	0.500
Respectful behavior of advisor with the students	0.90 ±2.60	0.90±2.50	0.500
The skill of advisor in the students´problem solving	1.40±1.70	1.20±2.10	0.110
Continuous training of advisor in student's problem solving	1.30±1.20	1.80±1.20	0.050
Friendly relationship between the student and advisor	0.90±1.90	1.00±2.30	0.070

**Table 3:** Mean scores of satisfaction about the performance of advisor in students of Para-medical Sciences school

**Performing the advisor project ** **Students´ satisfaction**	**Before (n=74) Mean±SD **	**After (n=74) Mean±SD**	p
Students’ satisfaction about the program of advisor ’presence at work	0.90±1.70	1.10±1.60	0.540
Devoting sufficient time for counseling	0.80±1.90	0.90±1.90	0.630
Providing access to advisor	1.20±1.50	1.10±1.20	0.140
Student’s benefit from the advice	1.30±1.30	1.30±1.50	0.140
Student’s Satisfaction about the impact of counseling	1.20±1.30	1.40±1.30	0.290
Students’ satisfaction about the advisor for solving the problem	1.20±1.30	1.30±1.60	0.090
Respectful behavior of advisor with the students	1.30±2.50	1.30±2.50	0.930
The skill of advisor in the students´problem solving	1.10±1.80	1.20±2.00	0.330
Continuous training of advisor in student's problem solving	1.30±1.30	1.70±1.40	0.060
Friendly relationship between the student and advisor	1.40±1.90	1.40±2.00	0.450

**Table 4:** Mean scores of satisfaction about the performance of advisor in students of Nursing and Midwifery school

**Performing the advisor project ** **Students´ satisfaction**	**Before (n=74) Mean±SD**	**Aftar (n=74) Mean±SD**	p
Students’ satisfaction about the program of advisor ’presence at work	0.90±1.90	0.90±2.10	0.300
devoting sufficient time for counseling	0.90±1.90	1.00±1.90	0.070
Providing access to advisor	1.10±1.80	1.00±2.60	0.010*
Student’s benefit from the advice	1.20±1.70	1.00±1.90	0.200
Student’s Satisfaction about the impact of counseling	1.30±1.00	0.90±1.20	0.430
Students’ satisfaction about the advisor for solving the problem	1.30±1.90	1.00±2.10	0.080
Respectful behavior of advisor with the students	1.10±3.00	0.90±3.00	0.440
The skill of advisor in the students´problem solving	1.10±0.90	1.10±1.10	0.120
Continuous training of advisor in student's problem solving	2.20±1.20	2.20±1.00	0.930
Friendly relationship between the student and advisor	1.30±2.50	1.10±2.60	0.350

## Discussion

The aim of this study was to compare the students’ satisfaction about the performance of advisors before and after performing the advisor project in Shahrekord University of Medical Sciences. The findings of study showed that the students report better conditions in items of "Having an advisor", "Knowing him as a source of educational information", "Knowing how to get help from a counselor ", "Making academic records", and " Asking the advisor about continuing the education", but there was not a significant difference in their satisfaction about the advisor´s performance before and after the advisors´ project. Although a similar study on examining the students´ satisfaction of advisor performance before and after the advisor project has not been conducted yet, but other studies, including the one performed in Ahvaz University of Medical Sciences, have reported that the students were not satisfied with the conditions of counseling and the advisor position as a resource of problem solving for them at the university has not acceptable (9). Similar findings have been reported at Kerman and Hamedan Universities of Medical Sciences (4, 6) that confirmed the findings of the present study. Other studies have also reported that the advice and guidance provided by the advisors has failed to be satisfactory for students (7, 10). In addition to these studies, there are other studies that reported an acceptable and satisfactory performance of the advisors (11).

In the present study, there was no significant difference in the satisfaction of students about the performance of advisors before and after the implementation of advisor’s project in students of medical, paramedical and health schools, but in students of nursing and midwifery school,  a significant difference was found only in the item of "providing access to advisor " before and after the advisors´ project; in this item, the students evaluated conditions better than after performing of the project. In one study, about 56% of the students were satisfied with their advisors and availability of the advisor; their information about the educational rules was the reasons for their satisfaction (12). This is in the same line with the findings of the present study. Another study has also reported that most of the students would rather the advisors have a executive position in their school (13). In one study conducted by Asadollahi et al, the viewpoints of advisors about the current desirable situation of academic counseling were examined. It is also reported that the advisors believed that their awareness of their responsibilities is at a moderate level (8). The findings of other studies imply less knowledge of advisors about the academic counseling and low awareness about their duties (14). A study conducted at the University of Urmia reported the moderate knowledge of supervisors about educational rules (15). A study that examined the students' reasons for lack of belief in advisors, reported that the students believed that they were able to solve their problems better than the advisors (16). The results of another study conducted in Isfahan University of Medical Sciences showed that few students regarded the advisors effective for their educational and personal problems (12). An Australian study reported that about half of the students in psychology school were not satisfied with their academic advisors and its main reason was the lack of knowledge about the advisor tasks (17). A study on counseling and guidance of students of universities in Turkey showed that the students’ referral to advisors was low (18). The results of all these studies are consistent with those of the present study.

Our study is unique in that it is the first to compare the students’ satisfaction before and after the advisor project and the findings indicated that the advisor project was not able to improve the students ‘satisfaction about the advisors’ performance. Other institutions have developed alternative strategies to enhance the student advising and meet other institutional needs. Sastre et al. (2010) reported that the Advisory College Program (ACP) was more effective in promoting student wellness and career counseling than the traditional one-on-one faculty advisor system (3). The University of Washington has created a college system consisting of 30 key faculties who not only develop one-on-one relationships with their assigned students but are also responsible for teaching clinical skills and professionalism throughout the 4 years (19). Finally, Coates et al. (2008) described a fourth-year medical student College Program based on career interests, designed to help students choose fourth-year electives, promote career mentoring opportunities, and improve the quality of the fourth year Education (20).

## Conclusion

It seems that the advisor project has failed to be satisfactory for students. There are several limitations in our study. First, our study was performed at a single institution, which may limit the ability to generalize our results. We were also unable to achieve 100% response rate, which raises questions as to the concerns or opinions of those students who were unable or chose not to complete the survey. However, we do believe that the striking difference noted between perceptions of advising before and after implementation of advisor project is not likely to be significantly diminished. Proper monitoring of the performing process, supporting the advisor and strengthening their position, informing them of their duties, and reducing their academic and research activities for further observation over the students under their control is suggested. 
